# Differential Gene Expression and Weighted Correlation Network Dynamics in High-Throughput Datasets of Prostate Cancer

**DOI:** 10.3389/fonc.2022.881246

**Published:** 2022-06-01

**Authors:** Taj Mohammad, Prithvi Singh, Deeba Shamim Jairajpuri, Lamya Ahmed Al-Keridis, Nawaf Alshammari, Mohd. Adnan, Ravins Dohare, Md Imtaiyaz Hassan

**Affiliations:** ^1^Centre for Interdisciplinary Research in Basic Sciences, Jamia Millia Islamia, New Delhi, India; ^2^Department of Medical Biochemistry, College of Medicine and Medical Sciences, Arabian Gulf University, Manama, Bahrain; ^3^Department of Biology, College of Science, Princess Nourah bint Abdulrahman University, Riyadh, Saudi Arabia; ^4^Department of Biology, College of Science, University of Hail, Hail, Saudi Arabia

**Keywords:** prostate cancer, precision oncology, target-propelled therapy, The Cancer Genome Atlas, weighted gene co-expression network analysis

## Abstract

Precision oncology is an absolute need today due to the emergence of treatment resistance and heterogeneity among cancerous profiles. Target-propelled cancer therapy is one of the treasures of precision oncology which has come together with substantial medical accomplishment. Prostate cancer is one of the most common cancers in males, with tremendous biological heterogeneity in molecular and clinical behavior. The spectrum of molecular abnormalities and varying clinical patterns in prostate cancer suggest substantial heterogeneity among different profiles. To identify novel therapeutic targets and precise biomarkers implicated with prostate cancer, we performed a state-of-the-art bioinformatics study, beginning with analyzing high-throughput genomic datasets from The Cancer Genome Atlas (TCGA). Weighted gene co-expression network analysis (WGCNA) suggests a set of five dysregulated hub genes (MAF, STAT6, SOX2, FOXO1, and WNT3A) that played crucial roles in biological pathways associated with prostate cancer progression. We found overexpressed STAT6 and SOX2 and proposed them as candidate biomarkers and potential targets in prostate cancer. Furthermore, the alteration frequencies in STAT6 and SOX2 and their impact on the patients’ survival were explored through the cBioPortal platform. The Kaplan-Meier survival analysis suggested that the alterations in the candidate genes were linked to the decreased overall survival of the patients. Altogether, the results signify that STAT6 and SOX2 and their genomic alterations can be explored in therapeutic interventions of prostate cancer for precision oncology, utilizing early diagnosis and target-propelled therapy.

## Introduction

Cancer is a highly complex, heterogeneous, and robust disease (1). It arises due to the failure at multiple levels in multicellular organisms. The failure at multiple levels includes genetic alterations, differential gene expression, metabolic disorders, and abnormal signal transduction processes at different signaling levels (receptor/intracellular/effector levels, conformational change, change in interactions) (2). The complexity of genomic profiles, expression patterns, and cellular interactions within the tumor microenvironment are the major challenges in understanding the disease mechanism (3). This complexity results from intratumoral heterogeneity (the substantial genetic diversity within tumors), where cancer cells have distinct molecular and phenotypic features established by different genetic alterations and environmental factors (4).

Prostate cancer is one of the leading causes of malignancy among men, with over 220,000 new cases diagnosed in 2015 in the United States only (5). Based on Globocan 2020 estimates, it is the second most common cancer (after lung cancer) in males and the third most common cancer worldwide, with over 1.4 million new cases and 3.75 lakh deaths in 2020 (6). Whereas the data regarding the factual incidences of prostate cancer in India is limited, still, it is the most prevalent cancer in men apart from skin cancer (7). For a prevalent malignancy like prostate cancer, relatively little is known about its etiology. Proven risk factors are inadequate to progressing age, family history, certain genetic mutations, and dysfunction of some androgen receptor (AR)-related genes. Also, a few lifestyle and environmental factors have been identified that may increase the risk of advanced prostate cancer, i.e., smoking, obesity, some nutritional factors, and race (6).

The available target-based therapeutic strategies and prostate-specific antigen (PSA)-based diagnostic approaches have come up with various off-target side effects and false positives in medical therapeutics of prostate cancer (8, 9). Considering this opportunity, many research groups have focused on identifying novel biomarkers and druggable targets of prostate cancer (10–15). However, due to the heterogeneity of cancerous profiles, it is tremendously unlikely to discover a single gene as a representative marker or druggable target in prostate cancer (16, 17). This heterogeneity may also underlie the high inconsistency of prostate cancer therapeutic diagnostic and clinical outcomes (18). Nonetheless, diagnostic kits made by combining multiple genes have been utilized to raise the prognostic power to detect prostate cancer, relapse, and survival after using traditional methods (19–22). Their commercial launch demonstrates the accomplishment of these diagnostic kits as ProMark (23), Oncotype DX (24), Prolaris (25), and Decipher (26). These kits may be upgraded by drawing from molecular classifications using DEGs from cancerous profiles, facilitating more precise outcomes, optimal therapies, and a better understanding of the disease.

Precision oncology, also known as biomarker-driven therapeutics, has significantly enhanced clinical outcomes in a little while (27). It highlights the efficacy of steering biomarkers and druggable targets associated with a poor prognosis and clinical outcomes affecting cancer patients’ healthy survival (28). Advancements in diagnostics, drug development, and biological research using modern approaches will greatly contribute to the medical therapeutics against prostate cancer under precision oncology (29). Genomic profiling of genetic alterations and analysis of expression patterns helps to understand prostate cancer’s complexity in different individuals (30). High-throughput next-generation sequencing (NGS) has facilitated the generation of molecular signatures of cancer. Here, differentially expressed genes (DEGs) between cancerous and non-cancerous profiles are abundant sources of putative biomarkers of cancer. Large-scale genomic characterization of cancerous profiles has offered vital new perceptions about the biological heterogeneity of prostate cancer and has the potential to discover novel biomarkers and druggable targets.

The high-throughput data of prostate cancer generated from different experimentations by various research laboratories across the world are publicly available at The Cancer Genome Atlas (TCGA) and the Genomic Data Commons (GDC) data portal (31). Genomic profiles in these repositories are progressively being exploited for precisely targeted therapeutic interventions in cancer research. Several studies have utilized these repositories to explore the genetic basis of prostate cancer and have found significant dysfunction of multiple genes (32–36). Notably, no comprehensive study has used the recent high-throughput data from the TCGA using an integrated bioinformatics approach to assess the genomic profiles of prostate cancer. Prostate cancer profiles show incredible heterogeneity. Some patients die of the metastatic condition within 2–3 years of diagnosis while others can survive for 10–20 years, probably reflecting the genomic diversity of profiles (37).

The genomic diversity can also be revealed by exploring gene regulatory networks in cancerous profiles. Gene regulatory networks are complex, and exploring their dynamics can discover key regulatory genes in complex diseases, including cancer (38). Studies on complex gene regulatory networks are based typically on clustering and identifying the high degree hubs, motif/modules from the network (39). These studies constructed from the high-throughput genomic datasets are used to understand better the key regulating genes in cancerous profiles and their roles in disease inception and progression (40–42). Integrated approaches, including network-based and DEGs analyses, are more helpful to optimize sensitivity and selectivity of diagnosis than investigating only a group of potentially unrelated genes in cancer. These approaches produce more robust outcomes, advance disease classification, and reveal new insights into the disease progression.

MicroRNAs (miRNAs) are small non-coding RNA molecules that function in RNA silencing and post-transcriptional regulation of gene expression. In contrast, transcription factors (TFs) are protein molecules, excluding RNA polymerase, that regulate the transcription of genes. miRNAs and TFs mutually regulate each other in a tightly coupled manner to form feed-forward loops (FFLs) or feed-back loops (FBLs) where a miRNA represses a TF, or a TF regulates a miRNA and both of them co-regulate a joint target (43). FFLs can be categorized into 3 types corresponding to their master regulators: miRNA-FFL, TF-FFL, and composite FFL (44). In a TF-FFL, TF is the master regulator which regulates its partner miRNA and their joint target, while in a miRNA-FFL, miRNA is the master regulator which represses its partner TF and their joint target (45). TF-FFL and miRNA-FFL merge to form a composite FFL, where TF and miRNA regulate/repress each other and their joint target (46).

We implemented an unbiased, comprehensive approach to get insights into prostate cancer’s biological heterogeneity and genomic characterization. A high-throughput dataset containing 459 cancerous and 50 normal profiles from TCGA was retrieved to analyze it using an integrated state-of-the-art computational approach (47). We have systematically analyzed these samples to find novel biomarkers and druggable targets for diagnostic, prognostic, and therapeutic delamination of prostate cancer. We carried out differential expression analysis and constructed the protein-protein interaction network (PPIN) to analyze DEGs. This integrated approach helps to reveal novel molecular markers and druggable targets for potential therapeutic approaches. Weighted gene co-expression network analysis (WGCNA) is an algorithm widely used to discover co-expressed modules correlated with phenotypes or traits based on expression data (48). Detection of meaningful densely correlated modules linked to specific clinical traits would be valuable for deducing tumor progression mechanisms and proposing novel hub targets that hamper vital signaling pathways. The differential gene expression analysis was followed by weighted Gene Co-expression Network (GCN) construction using a large-scale gene expression profile and trait-linked hub module detection. Then, we explored the alteration frequencies and performed survival analysis of the patients carrying genetic alterations in the identified candidate genes. This study provides a valuable insight into the differential expression and network dynamics of the genes associated with prostate cancer progression for the genomics community.

## Materials and Methods

**Figure 1:**
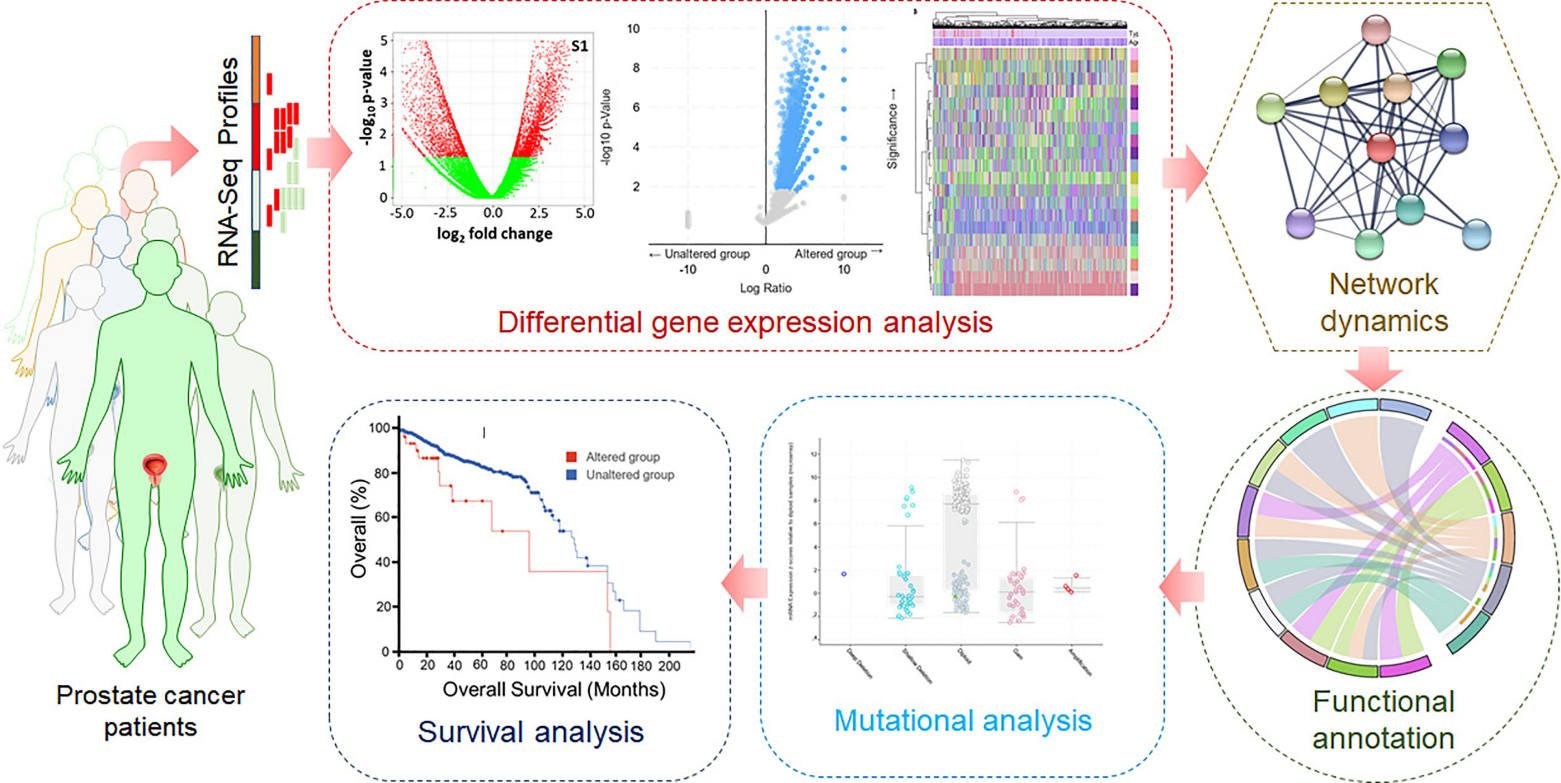
Graphical flowchart of the methodology.

High-throughput RNA-Seq data of prostate cancer patients were retrieved from the TCGA. First, the pre-processing was carried out for quality checks, batch correction, ID mapping, and normalization. For this purpose, various R packages such as sva, DESeq2, and edgeR were used. To identify the DEGs, the limma package was utilized. Visualization and data analysis, including the clustered heatmaps, were carried out using computational tools using ggplot2 and ComplexHeatmap packages. Weighted GCN analysis of significantly dysregulated genes was carried out to identify biomarkers and potential drug targets. A network biology approach was utilized to construct and analyze GCN, followed by PPIN construction and analysis. The gene ontology (GO) and pathway enrichment analyses were performed using Enrichr, a web-based bioinformatics resource for data mining (49). Here, monitoring the known signaling systems downstream in different biological processes associated with prostate cancer was carried out. Analyzing the network dynamics of significantly dysregulated genes will lead to identifying hub genes as biomarkers and potential druggable targets in prostate cancer. Mutational landscape and survival analyses of the cancerous profiles will contribute to understanding prostate cancer at the molecular level. A typical representation of the computational approach used in this study is illustrated in **Figure 1**.

### TCGA-PRAD RNA-Seq Data Extraction and Differential Expression Analysis

UCSC Xena browser (https://xenabrowser.net/) (50) was queried for extracting the messenger RNA (mRNA) HTSeq-counts (based on IlluminaHiSeq platform) and miRNA-Seq data of TCGA-prostate adenocarcinoma (PRAD) patient samples. Sample phenotype data such as age at diagnosis, weight, batch variables, and gender were also collected from Xena. Both these datasets were back log-transformed in R to obtain raw integer counts. To maintain an overall uniformity across samples, samples from both these datasets were then cross-checked with the mRNA-Seq and miRNA-Seq PRAD samples present in the TCGA-GDC data portal. Only primary solid tumor and solid tissue normal samples were retained in both datasets. ComBat-Seq model (51) available in sva (52) R package was applied on both datasets with known batches for correction. Low-count Ensembl IDs in the batch-corrected mRNA dataset were removed before normalization and log-transformation through variance stabilizing transformation (vst) using the DESeq2 package (53) in R. The Ensembl IDs in the mRNA dataset were mapped to their corresponding HUGO Gene Nomenclature Committee (HGNC) symbol(s) using the mapping file available from Xena. Expression of genes mapping values to multiple Ensembl IDs were averaged across all the samples to avoid redundancy. The edgeR package (54, 55) in R was applied to batch-corrected miRNA data for obtaining normalized (upper quartile) and log-transformed expression values. Limma package (56) in R was used to identify the DEGs (corresponding to a threshold of p-value<0.05 with |log_2_(fold change) | >0.1)) and DEmiRs (corresponding to a threshold of p-value<0.05). The criterion for low fold change was adopted to expand the maximum number of DEGs between tumor and normal sample groups. Since very few genes are differentially expressed at this fold change, making the fold change threshold more stringent would lead to nearly no DEGs or eliminate any important genes (57).

### Weighted Co-Expression Network Construction and Hub Module Identification

WGCNA R package (58) was utilized for weighted GCN construction and representative module genes classification correlated with clinical characteristics (59). The PRAD-specific DEGs and samples were passed through the *good Samples Genes* function to eliminate missing values. The samples were clustered after that to eliminate outliers. The clinical trait data (i.e., weight and age) of these PRAD-specific samples were also considered before identifying modules. Mean expression per array and the number of missing values per array were recorded. Any arrays with an excessive number of missing data were removed, followed by the deletion of any low variance genes. The *pickSoftThreshold* function assisted in selecting suitable soft-thresholding power (β) to which co-expression similarity will be raised for computing adjacency. β was chosen based on the approximate scale-free topology criterion. The weighted adjacency matrix was transformed into a topological overlap matrix (TOM), followed by a computation of corresponding dissimilarity (dissTOM) to reduce noise and false associations. The *hclust* function was utilized to generate a hierarchical clustering tree (dendrogram) of genes considering the dissTOM measure. A dynamic tree cut algorithm was incorporated to identify densely interconnected, highly co-expressed gene patterns (i.e., modules) from the branches of the tree. Module eigengene (ME) and dissimilarity measures between MEs were computed to merge the modules with highly co-expressed genes. ME dendrogram was checked based on Pearson correlation for merging multiple modules with comparable expression profiles. Correlation-based absolute module significance (GS) values (i.e., average gene significance of participating genes in a given module) with our trait of interest (i.e., weight) followed by module membership (MM) (correlation of the ME and the gene expression profile) for all were computed. The correlation of MM with GS was used to identify the most significant associations. The module having the significantly highest correlation with weight was chosen to be our hub module.

### PPIN Construction and Hub DEGs Selection

The DEGs present in our trait-linked hub module was subjected to PPIN construction using the STRING v11.0 (https://string-db.org/) web-based tool (60). The PPIN was formed at medium confidence (corresponding to interaction score >0.4) and afterward visualized using Cytoscape v3.8.2 (61). CytoHubba application (62) in Cytoscape was used to rank the top 10 DEGs corresponding to each centralities degree, namely - degree, stress, bottleneck, betweenness, closeness, and maximal clique centrality (MCC). The overlapping DEGs between these six ranked genesets were regarded as the hub DEGs.

### GO Term and Pathway Enrichment Analyses

GO term and pathway enrichment data for hub DEGs were compiled using GO-Biological Process (BP), GO-Molecular Function (MF), GO-Cellular Compartment (CC), and Kyoto Encylopedia of Genes and Genomes (KEGG) libraries available in the Enrichr database (63). GO terms and pathways corresponding to p-value <0.05 were statistically significant. Top 10 pathways and GO terms within this significant threshold were reported after that.

### PRAD-Specific 3-Node miRNA FFL Construction

Significant human TFs corresponding to score (p-value) <0.001 and regulating our hub DEGs were fetched from ChEA v3.0 database (64). Then, miRNAs (with a score >0.95 and binding only on 3’UTR region) repressing our hub DEGs and TFs (from ChEA) were extracted from miRWalk v3.0 (65) and starBase v2.0 (66) databases. These miRNAs were cross-checked with PRAD-associated DEmiRs, and only the overlapping ones were considered as final. All the interaction pairs (i.e., TF-DEG, miRNA-DEG, and DEG-TF) were altered to final TFs and miRNAs and merged to obtain a 3-node miRNA FFL (67).

### Mutational Analysis

Mutational frequencies in the identified candidate genes were explored in the TCGA dataset through cBioPortal (https://www.cbioportal.org/) (68). Each mutation was mapped on the 2D structure of the identified proteins, and their frequencies were noted. The domain organization of the proteins structures was generated to see each mutation in detail.

### Survival Analysis

To explore the impact of alterations in the identified candidate genes on the overall survival of patients with prostate cancer, we have performed the survival analysis in analytical procedures based on the TCGA dataset accessed from the cBioPortal. The data was plotted in Kaplan–Meier (KM) estimator (69) by applying the Logrank test P-value (70).

## Results and Discussion

### TCGA-PRAD RNA-Seq Data Extraction and Differential Expression Analysis

PRAD-specific mRNA and miRNA datasets comprised a total of 509 samples (459 tumor and 50 healthy normal samples) following the search criteria specified. 51923 IDs were left after deleting low count Ensembl IDs in the batch-corrected mRNA dataset. After normalization and log transformation using DESeq2, mapping of IDs to their corresponding genes was performed. Lastly, 50711 unique genes were left after averaging expression values of duplicate genes. We obtained 1097 miRNAs after filtering the ones with low CPM values, followed by TMM normalization and log transformation using edgeR. Now both these miRNA and mRNA datasets were subjected to limma where we obtained 1571 DEGs and 49 DEmiRs following the threshold above, i.e., p-value<0.05 with |log_2_(fold change) | >0.1 (for DEGs) and p-value <0.05 (for DEmiRs). A total of 844 and 727 DEGs were bifurcated as up and downregulated, respectively. In addition, 6 and 43 DEmiRs were bifurcated as up and downregulated, respectively.

### Weighted Co-Expression Network Construction and Hub Module Identification

**Figure 2:**
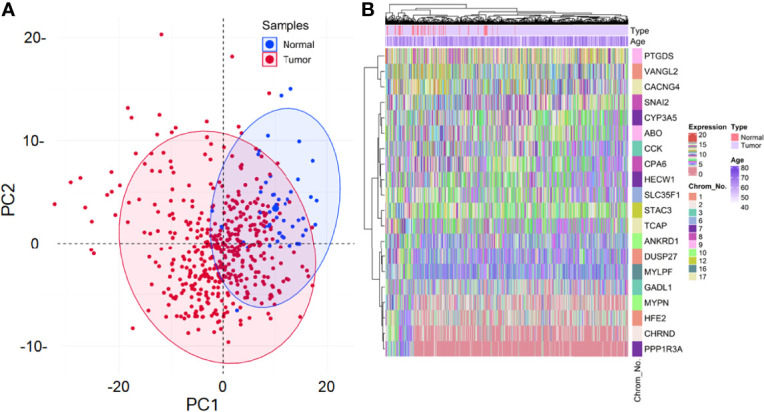
**(A)** Principal component analysis (PCA) plot shows the expression variability of 221 DEGs across all samples. Each point in the plot signifies the relative expression value of all DEGs dimensionally reduced to sample type leading to distinct cluster formations. Blue and red points signify normal and tumor samples, respectively. The percentage of total variation accounted for by the 1st (30.2%) and 2nd (9.9%) principal components are shown on the x and y axes, respectively. **(B)** Annotation heatmap showing the expression distribution of top 10 up and downregulated PRAD-specific DEGs. Cluster dendrograms representing Euclidean distance-based hierarchical clustering for both rows and columns are presented along the left and top sides of the plot. Sample type (red for normal and violet for tumor) and age annotation bars are presented at the top of the heatmap. The location of each gene on its respective chromosome number is presented in the right panel as the row annotation bar (multi-colored bands).

From 1571 DEGs, the ones with non-protein-coding type and identified as outliers were removed, leaving 221 DEGs for further analyses. Also, 17 samples were identified as outliers from the sample clustering dendrogram and were removed by cutting the branch at height=24. The clinical information associated with these 492 samples can be found in **Supplementary Table S1**. None of the 221 DEGs had low-variance expression. **Figure 2A** shows the principal component analysis (PCA) plot exhibiting the expression distribution of these DEGs across all samples. The expression variability of all the DEGs was dimensionally reduced to sample type leading to distinct cluster formations. **Figure 2B** shows the expression heatmap of the top 10 up and downregulated DEGs. Within all these 20 DEGs, CCK [log_2_(fold change)=0.79] and TCAP [log_2_(fold change)=-0.57] were most up and downregulated ones. The sample type and age annotation bars were placed at the top of the heatmap. The age of samples varied from 41 to 77, with the maximum number of patients (i.e., 34) having age 66. The chromosome annotation bar was shown on the right, where maximum genes were present on chromosome number 1 (i.e., VANGL2, DUSP27, HFE2) and 7 (i.e., HECW1, PPP1R3A, CYP3A5), respectively. β=5 was chosen (corresponding to scale-free *R*^2^*=*0.85) as the soft-thresholding power for constructing a weighted co-expression network. **Supplementary Figures S1A, B** shows plots for β in consideration with scale-free topology criteria.

**Figure 3:**
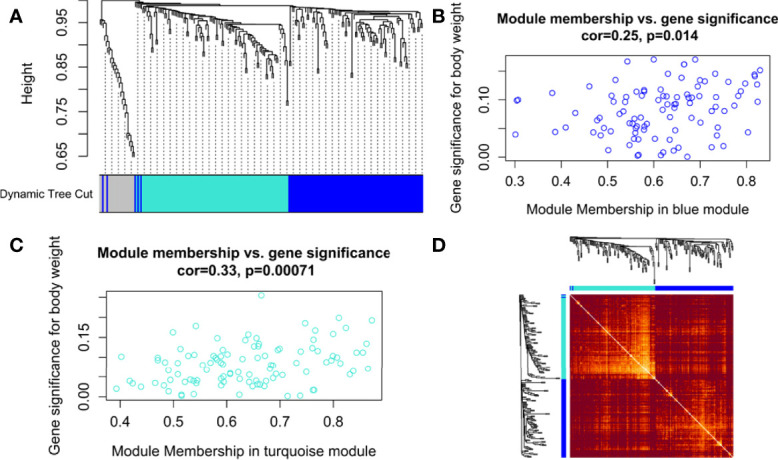
**(A)** Hierarchical clustering dendrogram of 221 PRAD-specific DEGs clustered based on the dissimilarity measure (dissTOM) along with 3 color-coded modules (obtained using Dynamic Tree Cut). The modules contained highly similar expression profiles with the following sizes: turquoise (102), blue (97), and grey (22). Scatterplots show a significant (p-value <0.05) correlation of Gene significance (GS) for weight with module membership (MM) in **(B)** blue and **(C)** turquoise modules. **(D)** Topological Overlap Matrix (TOM) plot of the weighted gene co-expression network representing TOM among blue and turquoise module genes. Hierarchically clustered gene dendrograms and module assignments are presented along the top and left sides of the plot. Lighter and darker shades signify lower and higher overlap among the genes. Dark-colored blocks along the diagonal represent modules.

The hierarchical clustering tree and dynamic tree cut algorithm revealed three color-coded modules (i.e., blue, turquoise, and grey), as shown in **Figure 3A**. There was no need for merging these modules due to the low merging height observed in the ME dendrogram. **Supplementary Figure S1C** shows an association of MEs for each module with weight and age as the color-coded table. **Supplementary Figure S1D** shows a Barplot of GS correlated with weight across module genes. The blue (module significance=0.082) and turquoise (module significance=0.081) colored modules were the most promising. **Supplementary Figure S2A** compares weighted and Pearson correlations. The standard method (simple Pearson correlation) ignores ME information. Multi-dimensional scaling (MDS) plot of all modules in 3 scaling dimensions is shown in **Supplementary Figure S2B**. Since the grey module consists of unassigned genes, we discarded it for further analysis. **Figures 3B, C** shows a scatterplot of GS for weight to MM in blue and turquoise modules. **Figure 3D** shows the gene co-expression network as a heatmap plot. It depicts TOM among the blue and turquoise module genes. A significantly high correlation between GS and MM is noticed in the turquoise (cor=0.33) as compared to the blue (cor=0.25) module. **Supplementary Figures S2C, D** shows a significant relationship between MM (raised to β=5) and intramodular connectivity in blue and turquoise-colored modules. Clearly, the turquoise module (cor=0.74) had a stronger relationship than the blue module (cor=0.58) between MM and intramodular connectivity. The turquoise module was selected as our hub module based on all these results.

### PPIN Analysis and Hub DEGs Selection

**Figure 4:**
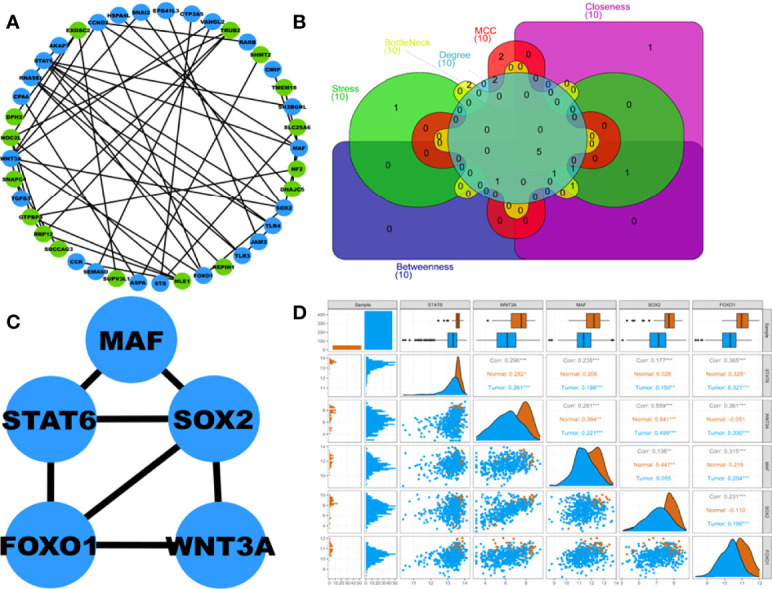
**(A)** Protein-protein interaction (PPI) network comprising 41 nodes and 48 edges corresponding to STRING interaction score>0.4. Blue and green nodes represent up and downregulated proteins, respectively. **(B)** Venn plot showing 5 overlapping hub DEGs within 6 centrality-based ranked genesets. The Venn plot’s green, red, cyan, magenta, yellow, and violet areas represent stress, MCC, degree, betweenness, bottleneck, and closeness centrality-based ranked genesets. **(C)** Highest-scoring PPI hub cluster comprises 5 nodes and 7 edges. **(D)** Pairwise scatter plot showing the associations amongst these 5 hubs upregulated DEGs. The upper triangular section represents the Spearman correlation coefficients between these DEGs and expression boxplots for each DEG. The lower triangular section represents the scatterplot and histogram distribution between these DEGs. The diagonal consists of kernel densities for each DEG. Significant levels at 0.05, 0.01, and 0.001 are represented by *, **, and ***, respectively.

A total of 41 out of 102 DEGs within the turquoise hub module participated in the PPIN corresponding to a STRING interaction score >0.4. The PPIN, as shown in **Figure 4A**, comprises 41 nodes and 48 edges. **Figure 4B** shows the Venn plot of six gene sets ranked based on each centrality (i.e., degree, stress, bottleneck, betweenness, closeness, MCC) within the PPIN. The 5 overlapping hub DEGs within these gene sets were STAT6, WNT3A, MAF, SOX2, and FOXO1 (**Supplementary Table S2**). **Figure 4C** shows the PPI subnetwork comprising these 5 hub DEGs as nodes and linked by 7 edges. A pairwise scatter plot matrix exhibiting association among these five upregulated hub DEGs is shown in **Figure 4D**. The highest correlation of 0.559 was observed within the plot between SOX2 and WNT3A, while the lowest correlation of 0.138 was observed between SOX2 and MAF.

### GO Term and Pathway Enrichment Analyses

**Figure 5:**
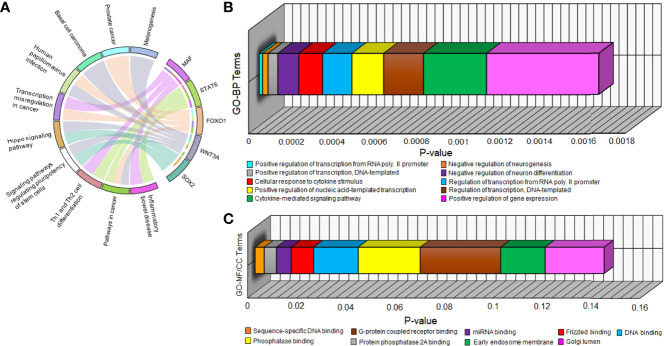
**(A)** Chord plot showing the relation of 5 hub DEGs (on right semicircle track) with 10 significant pathways (on left semicircle track) *via* colored edges. The edges initiate from unique colored strips present on the right semicircle (indicating genes) and converge to unique colored strips present on the left semicircle (indicating pathways). Three-dimensional horizontal bar plots show top 10 significant **(B)** GO-BP and **(C)** GO-MF/CC terms on the y-axis regarding p-values on the x-axis. Every unique colored block signifies a different GO term.

All 5 hub DEGs participated in the top 10 significant pathways with inflammatory bowel disease (p-value=1.03 x 10^-4^) being the most significant. A chord plot displaying the association of these 5 hub DEGs with 10 significant pathways is shown in **Figure 5A**. Interaction edges in the chord plot display that both WNT3A and FOXO1 were present in the maximum number of pathways (i.e., 5 pathways) whereas SOX2 was present in the minimum number of pathways (i.e., 2 pathways). Within the abovementioned significant threshold (i.e., p-value<0.05), top 10 BP, top 8 MF, and top 2 CC terms were screened. **Figures 5B, C** shows 3-dimensional stacked bar plots representing these top significant GO-BP, MF, and CC terms (on the y-axis) with respect to their values (on the x-axis). The most significant GO-BP, MF, and CC terms were positive regulation of transcription from RNA polymerase II promoter (p-value=1.55 x 10^-5^), protein phosphatase binding (p-value=2.07 x 10^-4^), and early endosome membrane (p-value=1.81 x 10^-2^). SOX2 was a part of the highest number of GO-BP terms (i.e., 10). SOX2 and FOXO1 were a part of the highest number of GO-MF terms (i.e., 4).

### PRAD-Specific 3-Node miRNA FFL Analysis

**Figure 6:**
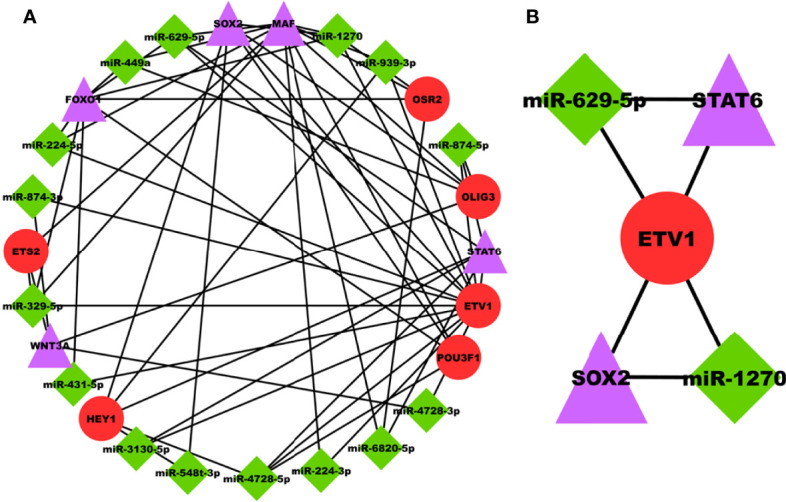
**(A)** PRAD-specific 3-node miRNA FFL network comprising 26 nodes and 58 edges. Red circular nodes represent TFs, green diamond nodes represent PRAD-specific miRNAs, and magenta triangular nodes represent hub DEGs. **(B)** PRAD-specific highest-order subnetwork FFL motif comprising one joint TF (ETV1), two miRNAs (miR-1270 and miR-629-5p), and two hub DEGs (SOX2 and STAT6).

miRNAs are majorly found to be involved in the development and progression of prostate cancer and are appealing as key regulators in disease progression. The PRAD-specific 3-node miRNA FFL as shown in **Figure 6A**, comprises 26 nodes and 58 edges. All the FFL edges, 14, 21, and 23, belonged to TF-DEG, miRNA-TF, and miRNA-DEG pairs. Whereas 15, 5, and 6 nodes belonged to miRNAs, DEGs, and TFs, respectively. Within the TF-DEG pair, both SOX2 and MAF were regulated by a maximum number of TFs (i.e., 4), while both OLIG3 and POU3F1 regulated the maximum number of DEGs (i.e., 3). Within the miRNA-TF pair, ETV1 was repressed by the maximum number of miRNAs (i.e., 14), while miR-4728-5p repressed the maximum TFs (i.e., 3). Lastly, within the miRNA-DEG pair, MAF was repressed by the maximum number of miRNAs (i.e., 8) while miR-1270 and miR-629-5p repressed the maximum number of DEGs (i.e., 3). Overall FFL analysis revealed a highest-order FFL subnetwork motif, as shown in **Figure 6B**, comprising one joint TF (ETV1), two miRNAs (miR-1270 and miR-629-5p), and two hub DEGs (SOX2 and STAT6) joined with 6 interaction edges. The FFL analysis disclosed both SOX2 and STAT6 as the most potential PRAD-specific hub DEGs. The analyses revealed that SOX2 and STAT6 represent themselves as potential candidates in prostate cancer profiles explored under this study. The limited specificity of the available tests conveys a requirement to develop novel and better diagnostic tools. In modern science, bioinformatics approaches provide significantly better biomarkers with improved features that can not only be used for diagnostic purposes but also for staging, evaluating aggressiveness and therapeutic procedures. The poor prognostic value of PSA and available biomarkers in clinical practices does not support timely therapy management and intervention. The study suggests that SOX2 and STAT6 can be explored as novel biomarkers and potential targets in target-propelled therapy against prostate cancer.

### Targeting SOX2 and STAT6 in Prostate Cancer

SOX2 [SRY (sex-determining region Y)-related high-mobility box 2] is a crucial transcription factor that plays a vital role in tissue homeostasis, embryonic development and maintenance of undifferentiated embryonic stem cells (71). SOX2 amplification, typically in pairs with abnormally increased expression, has been found in human cancers, i.e., breast, prostate, lung, colon, and ovarian (71). Overexpression of SOX2 endorses cancer progression by stimulating cell proliferation, invasion, migration, and sphere formation. It is causally linked with developing the resistance of cancer cells to chemotherapy, radiotherapy and targeted therapy in various cancers (72). It is repressed by AR signaling, promoting castration-resistant prostate cancer, where the percentage of SOX2-positive tumors increases with Gleason Score and metastasis (73). Loss of SOX2 expression in the prostate cancer cell line resulted in cell growth inhibition (73). In many cell lines studies, SOX2 and some other genes endorse cell proliferation and survival and destruct normal differentiation processes, hallmarks of cancer progression (71, 74–76). Thus, our study, along with the reports mentioned above and some others, validates SOX2 as a significant marker and to be used as a promising target in prostate cancer (77, 78).

STAT6 (signal transducer and activator of transcription 6) is another transcription factor that plays a vital role in regulating cell proliferation, differentiation, apoptosis and angiogenesis and organizing the epigenetic setting of immune cells (79). It is causally associated with cancer development, progression, metastasis, resistance to treatment; thus is of interest in cancer biology (80). High expression of STAT6 is associated with poor clinical consequences in cancer patients (81). Studies have reported that STAT6 signaling is vital for IL-4- and IL-13-induced epithelial-mesenchymal transition and aggressiveness of colorectal cancer cells (82, 83). Amplification of STAT6 has been found in many dedifferentiated liposarcomas and solitary fibrous tumors, leading to a NAB2-STAT6 oncogene fusion (84). A few studies targeting STAT6 signaling reported reduced tumor growth in gastric cancer (85), breast cancer (86), and prostate cancer (87). In a scientific study, STAT6 expression was higher in prostate cancer tissues than in normal tissues (88). Here, miRNA−135a induced prostate cancer cell apoptosis *via* targeting STAT6 (88). A study suggests that STAT6 acts as a survival factor in prostate cancer and regulates the genetic transcriptional driver responsible for cancer progression (80). Here, STAT6 expression was noticeably associated with high histological grades of prostate cancer and tumor size (80). Another finding suggests that STAT6 interaction with Annexin A2 could potentially affect prostate cancer’s metastasis process (89). All these studies associated with our study suggest that STAT6 might be a potential target for prostate cancer.

### Mutational Frequencies in SOX2 and STAT6

**Figure 7:**
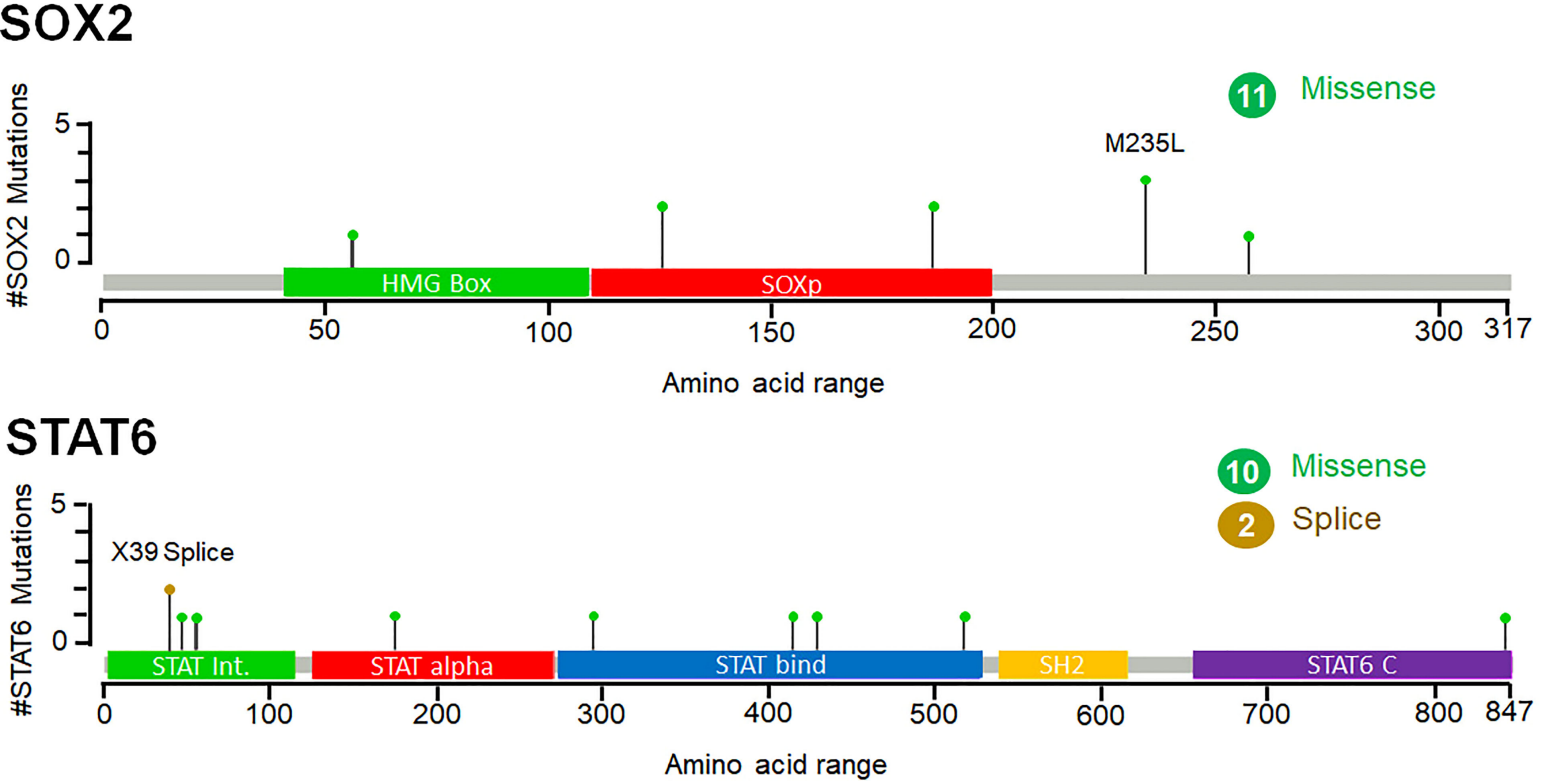
Frequency of point mutations and their types of SOX2 and STAT6 in prostate cancer. The figure was generated through the cBioPortal based on the TCGA datasets. Green lollipops represent the missense mutations in SOX2 and STAT6.

While exploring the TCGA datasets in cBioPortal, it was observed that 11 and 12 mutations were located within different domains of SOK2 and STAT6, respectively. These mutations were mapped with their frequency in various cancerous profiles (**Figure 7**). Overall, the somatic mutation frequencies in SOX2 and STAT6 were estimated to be 0.2%, which are adjacent to the regions critical for the functional activity of the proteins. Mutations in SOX2 (N187T, M235L, T126K, R56W, N187T, S258Y) and STAT6 (X39_splice, R294Q, D429G, W517C, P175S, A47T, A55S, R294Q, D429G, A843V, V414I) were distributed throughout the structure; among them, a missense mutation M235L in SOX2 and a splice at X39 in STAT6 was found with maximum occurrence (**Figure 7**). These mutations might be associated with the structural alterations in SOX2 and STAT6 and thus their dysfunction leading to prostate cancer progression. We have also compared the genetic alteration in the elucidated biomarkers with the published biomarkers in prostate cancer patients. We have generated an OncoPrint showing the genomic alterations in prostate cancer known biomarkers, KLK3 (PSA), PCA3, and DLX1 and elucidated biomarkers, SOX2 and STAT6 (**Figure S3**). While querying 8259 patients/8549 samples in TCGA, we found genomic alterations in KLK3, PCA3, DLX1, STAT6 and SOX2 in 46, 41, 60, 40 and 145 samples, respectively (TCGA accessed on 28 March 2022). The highest genetic alterations were found in SOX2, one of the elucidated biomarkers in our study, i.e., 1.8%, mainly by amplification and deep deletion.

### Survival of the Patients

**Figure 8:**
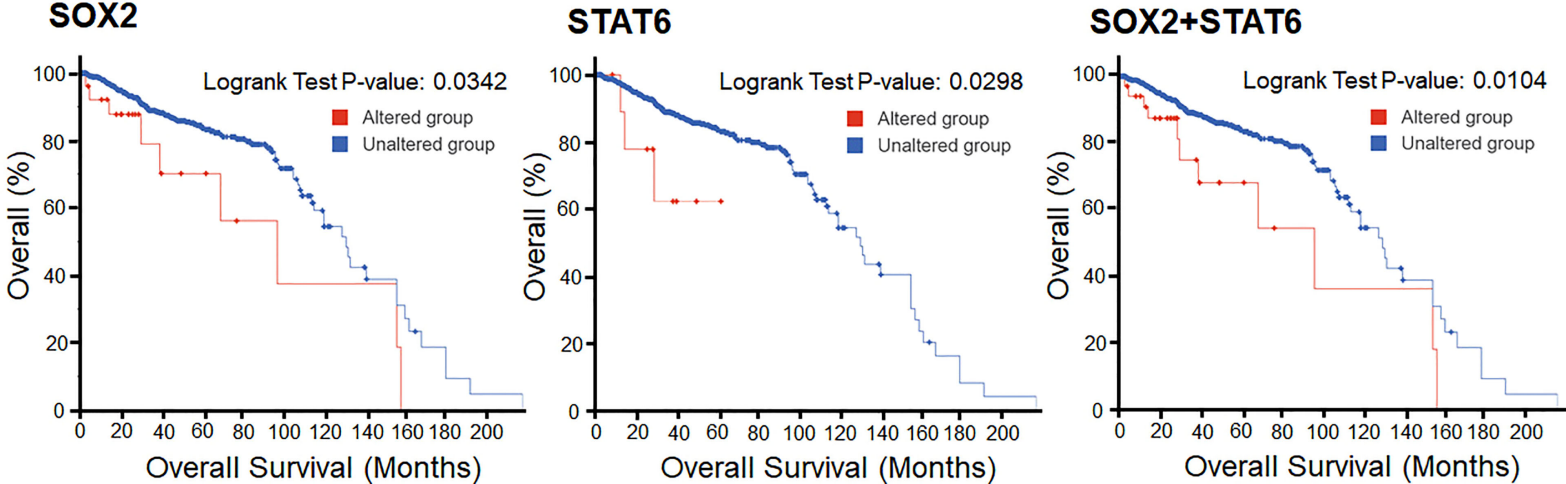
Overall Kaplan-Meier survival estimation of prostate cancer patients in the TCGA dataset. The lower panel shows the tabular status of the Kaplan-Meier survival estimation.

The survival analysis of prostate cancer patients shows that the alterations in SOX2 and STAT6 are efficiently responsible for the decrement in the overall survival of the individuals. In KM estimation survival analysis, the patients’ survival was effectively reduced where 9 patients were deceased out of 27 cases where SOX2 was altered. This altered group shows 97 months of median survival. At the same time, 133 patients were deceased out of 954 cases where SOX2 was not altered and showed 131 months of median survival. In contrast, 3 patients were deceased out of 22 cases where STAT6 was altered. At the same time, 140 patients were deceased out of 971 cases where STAT6 was not altered and showed 131 months of median survival. Overall, 11 death events were observed out of 36 cases where both genes, SOX2 and STAT6, were altered. This altered group shows 97 months of median survival. At the same time, a total of 131 death events were observed out of 945 cases where the selected genes were not altered and showed 131 months of median survival. However, the KM estimation of progression-free survival data is not enough to make an effective conclusive remark. Still, the effect of the genomic alterations as a reduced survival rate can be observed from the plots (**Figure 8**).

## Conclusions

Understanding the genomic level complexity is one of the major challenges in cancer research. This work provides a better understanding of prostate cancer at the genomic level by finding novel candidate genes and genomic lesions responsible for its onset and progression. In addition, expression profiling of DEGs in prostate cancer at the molecular level was utilized for gene pathway analysis and finding biomarkers for therapeutic applications. A TCGA PRAD dataset was analyzed using an integrated bioinformatics approach, including WGCNA. Initially, a set of 5 hub genes (MAF, STAT6, SOX2, FOXO1, and WNT3A), and later 2 most dynamic hub genes, STAT56 and SOX2, were identified. Both genes are significantly enriched in various biological pathways, primarily linked to the cell cycle process, chemokine-mediated signaling pathways in prostate cancer. The point mutations and their types in SOX2 and STAT6 in prostate cancer patients shows significant mutation frequency. The KM survival shows that the patients with prostate cancer held STAT6 and SOX2 alterations, linked to the decreased survival of the patients. We described identifying novel biomarkers followed by DEGs and WGCNA analyses. Our study provides a deeper insight into the understanding of heterogeneity and underlying molecular trials in prostate cancer. This work’s social relevance and applications may reflect in early detection and diagnosis of prostate cancer, personalized treatments, a selection of suitable model organisms, drug development, and many more.

## Data Availability Statement

The original contributions presented in the study are included in the article/**Supplementary Material**. Further inquiries can be directed to the corresponding author.

## Author Contributions

Conceptualization and study design, TM, PS, DJ, and MH; Methodology TM, LA-K, NA, and PS; resources, MA, LA-K, MH, and RD; writing—original draft preparation, TM, DJ, and PS; writing—review and editing, TM, LA-K, NA, RD, and MH; supervision, LA-K and MH; project administration, RD, and MH; funding acquisition, LA-K, and MH. All authors contributed to the article and approved the submitted version.

## Conflict of Interest

The authors declare that the research was conducted in the absence of any commercial or financial relationships that could be construed as a potential conflict of interest.

## Publisher’s Note

All claims expressed in this article are solely those of the authors and do not necessarily represent those of their affiliated organizations, or those of the publisher, the editors and the reviewers. Any product that may be evaluated in this article, or claim that may be made by its manufacturer, is not guaranteed or endorsed by the publisher.
