# Supplementary material for: Differential Gene Expression and Weighted Correlation Network Dynamics in High-Throughput Datasets of Prostate Cancer
Source: Front Oncol. 2022 Jun 1;12:881246. doi: 10.3389/fonc.2022.881246 (PMC9198298; doi:10.3389/fonc.2022.881246)
Supplement: Supplementary file 1 [file Presentation_1.pdf]

Supplementary data

Differential gene expression and weighted correlation network dynamics in high-throughput datasets of prostate cancer

Taj Mohammad[1], Prithvi Singh[1], Deeba Shamim Jairajpuri[2], Lamya Ahmed Al-Keridis[3], Nawaf Alshammari[4], Mohd. Adnan[4], Ravins Dohare[1], and Md Imtaiyaz Hassan[1,*]

[1]Centre for Interdisciplinary Research in Basic Sciences, Jamia Millia Islamia, New Delhi-110025, India.

[2]Department of Medical Biochemistry, College of Medicine and Medical Sciences, Arabian Gulf University, Manama, Bahrain.

[3]Department of Biology, College of Science, Princess Nourah Bint Abdulrahman University, P.O. Box 84428, Riyadh, 11671, Saudi Arabia.

[4]Department of Biology, College of Science, University of Hail, P.O. Box 2440, Hail, Saudi Arabia.

**To whom all correspondence should be addressed,*

Md. Imtaiyaz Hassan, PhD, FRSB, FRSC.
Assistant Professor
Centre for Interdisciplinary Research in Basic Sciences
Jamia Millia Islamia, Jamia Nagar
New Delhi 110025, India
Telefax: +91-11-2698-3409
E-mail: mihassan@jmi.ac.in

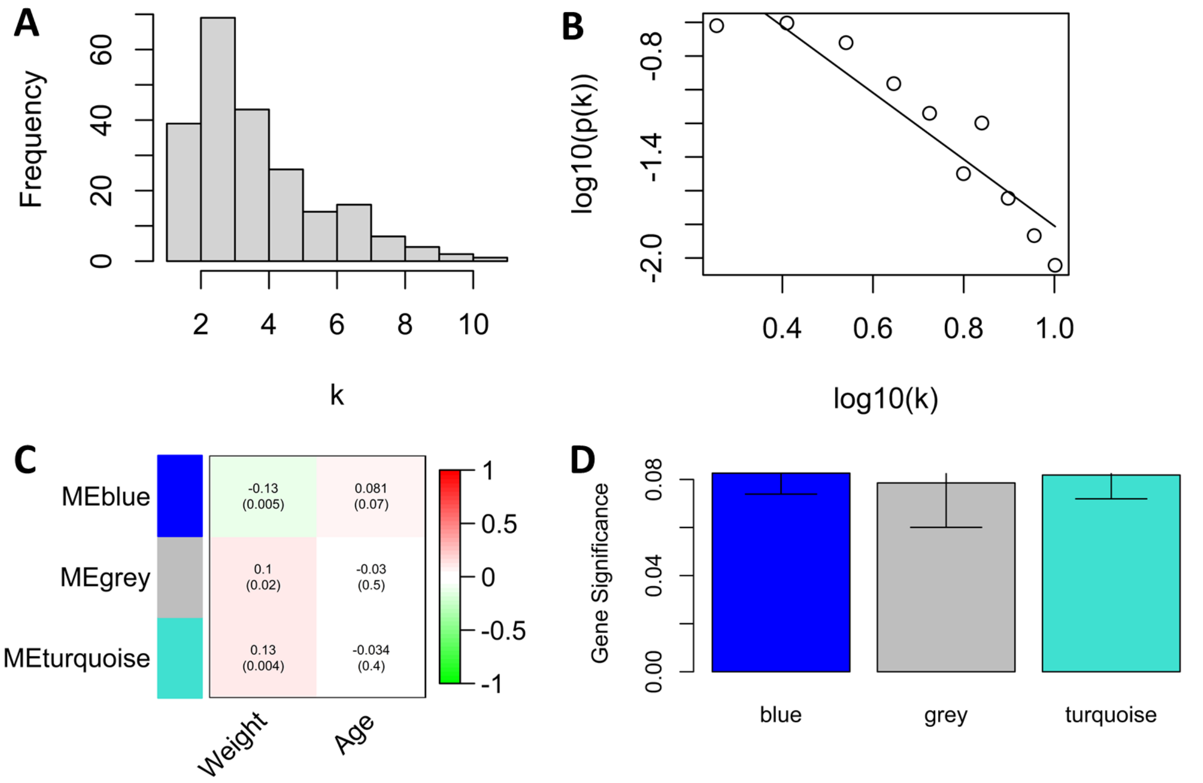

Figure S1: ***(A)*** *Histogram of network connectivity distribution when* $\beta = 5$*.* ***(B)*** $log_{10}(k)$ *vs* $log_{10}(p(k))$ *plot of the same histogram where the scale-free topology is depicted by the approximate straight-line relationship (high* $\boldsymbol{R^2 = 0.85}$*) and a negative value of slope (*$\boldsymbol{slope = -1.98}$*).* ***(C)*** *Color-coded table showing the association of Module Eigengenes (MEs) for each module with respect to weight and age. Each row corresponds to a ME and column to weight and age traits. Each cell consists corresponding correlation and p-values. The color-coding of the table varies with respect to correlation values as given in the vertical-colored legend bar at extreme right.* ***(D)*** *Barplot of module significance aka "mean GS" across all genes in the module correlated with respect to weight. Both blue and turquoise modules were most promising.*

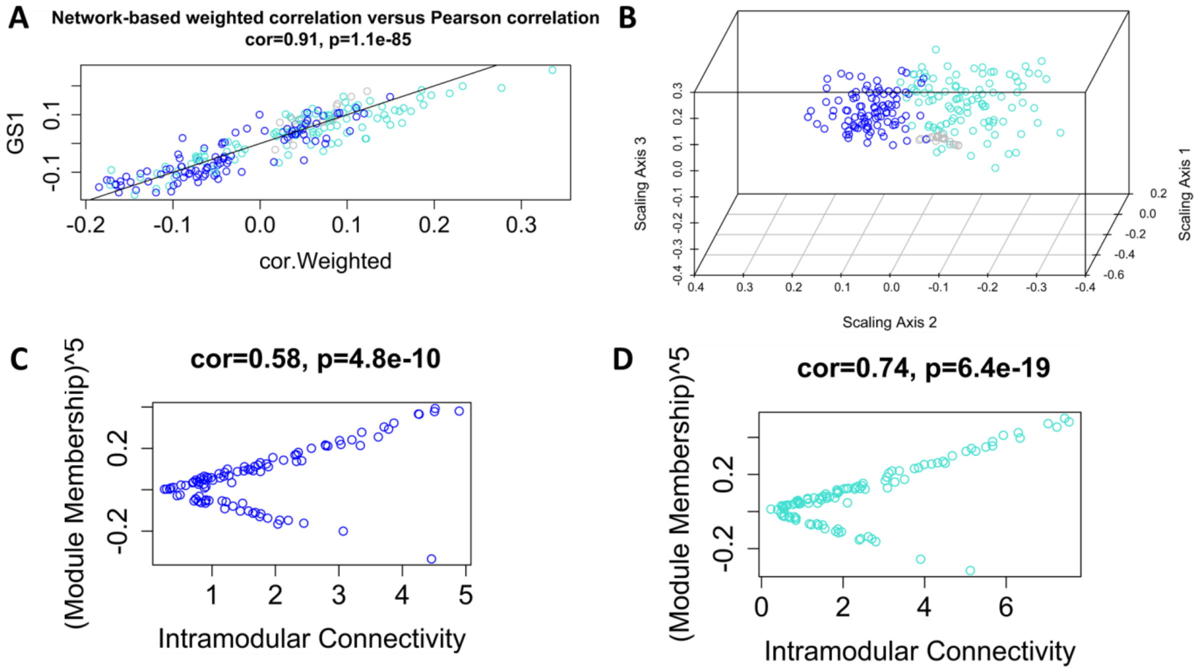

Figure S2: (A) *Weight-based GS in the training set (y-axis) plotted against the network-based significance (x-axis). WGCNA amplifies correlations of genes that are members of outcome related modules.* ***(B)*** *3-Dimensional Multidimensional Scaling (MDS) plot where each colored circle denotes a single gene belonging to the module of the corresponding color. MDS takes dissimilarity (dissTOM) as input and returns a set of points such that the distances between them are approximately equal to dissTOM. The 3 scaling axes across 3-dimensions represent the corresponding range of distances. Correlation plots of intramodular connectivity aka "*$\boldsymbol{k.in}$*" (across x-axis) vs MM for* ***(C)*** *blue and* ***(D)*** *turquoise modules. The color of dots indicate the genes within that particular colored module. After raising MM to* $\boldsymbol{\beta} = \mathbf{5}$*, it is highly correlated with* $\boldsymbol{k.in}$ *for both the modules.*

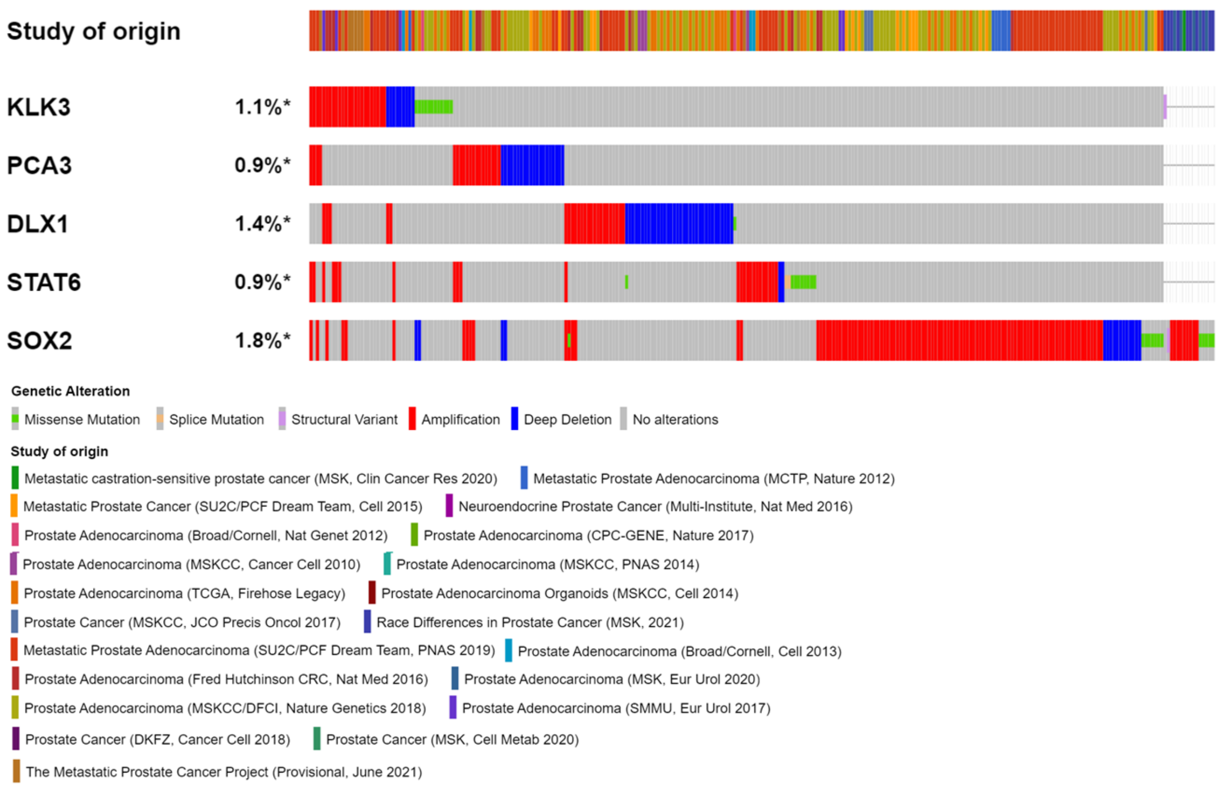

Figure S3: *OncoPrint showing the genetic alterations in prostate cancer known biomarkers, KLK3 (PSA), PCA3, and DLX1 and elucidated SOX2 and STAT6. While querying 8259 patients / 8549 samples in The Cancer Genome Atlas (TCGA), we found genomic alterations in KLK3, PCA3, DLX1, STAT6 and SOX2 in 46, 41, 60, 40 and 145 samples, respectively (TCGA accessed on 28 March 2022). The highest genetic alterations were found in SOX2, i.e., 1.8%, mainly by amplification and deep deletion.*

Table S1: Prostate cancer patients clinical information from TCGA.

Characteristics	Samples (n = 492)	Tumor (n = 442)	Normal (n = 50)
**Age**			
< 65	332	299	33
≥ 65	160	143	17
**Race**			
White	410	367	43
Black	59	52	7
Asian	11	11	0
American Indian	1	1	0
Not Reported	11	11	0
**Pathological T stage**			
T2	206	188	18
T3	270	240	30
T4	11	9	2
Not Reported	5	5	0

Table S2: Increased expression of the selected genes in prostate cancer samples from TCGA.

Gene Symbol	Name	$\log_2(\text{fold change})$	p-value
STAT6	Signal Transducer and Activator of Transcription 6	0.18	0.027
SOX2	SRY-Box Transcription Factor 2	0.43	0.042
WNT3A	Wnt Family Member 3A	0.40	0.049
MAF	MAF BZIP Transcription Factor	0.27	0.034
FOXO1	Forkhead Box 1	0.18	0.038
